# Natural Products and Their Derivatives with Antibacterial, Antioxidant and Anticancer Activities

**DOI:** 10.3390/antibiotics10010070

**Published:** 2021-01-13

**Authors:** Thu V. Vuong

**Affiliations:** Department of Chemical Engineering and Applied Chemistry, University of Toronto, 200 College Street, Toronto, ON M5S 3E5, Canada; thu.vuong@utoronto.ca

Natural products and their derivatives have been commonly used in our daily life, as they play important roles in boosting immune systems and fighting diseases. However, novel bioactive compounds are continuously in demand due to a rise of antibiotic resistance, cancer and infectious outbreaks, including recent pandemics (e.g., COVID-19). To contribute to that quest, this Special Issue showcases 13 papers, highlighting recent advances in the isolation and characterization of natural products and their derivatives with antibacterial, antioxidant and anticancer properties. 

The majority of natural products are produced from plants, and one of the largest groups of plant-derived products is flavonoids, as more than 13,000 plant flavonoids have been isolated and identified [1]. These plant-derived compounds have remarkable pharmaceutical and medical applications, particularly in preventing diseases. Among plant flavonoids, pectolinarin and its aglycone, pectolinarigenin, are identified as the major constituents in many medicinal herbs around the world. A review on the isolation, pharmacological aspects and therapeutic potential of these two flavonoids was written by Cheriet et al. [1], providing a comprehensive summary of our understanding of these secondary metabolites. Another widely distributed group of products in the plant kingdom is that of stilbenoids. Mattio et al. [2] reviewed recent achievements in the study of resveratrol and other natural stilbenoids as antimicrobial agents. The authors provided a thorough overview of the sources, chemical structures (particularly structural modifications), and the mechanism of action of stilbenoids. 

Many drug discovery campaigns have been launched to explore potent bioactive molecules from plants and microorganisms. Tran et al. [3] successfully isolated two prenylated acetophenones from the fruit of the Australian endemic plant *Acronychia crassipetala.* Their structures were analyzed by high-resolution mass spectrometry, as well as 1D (H-NMR and C-NMR) and 2D nuclear magnetic resonance spectroscopy (COSY, HSQC, HMBC and NOESY). The research group revealed that one compound is new while the other shows increased inhibition against *Staphylococcus aureus,* compared to the antibiotic chloramphenicol [3]. Using similar mass spectrometry and NMR techniques, Primahana et al. [4] elucidated five unprecedented β-carboline derivatives from a rare actinobacterium. One of these isolated molecules displayed moderate antifungal activity, and the other exhibited significant cytotoxic activity against a human lung carcinoma cell line [4]. Similarly, Anoumedem et al. [5] revealed the structures of two new tetracyclic polyketides that were isolated from an endophytic fungus. This study further extends the range of compounds that are produced by the genus *Simplicillium.* Fyhrquist et al. [6], who also followed the bioactivity-guided fractionation approach, used water and solvents (methanol, butanol and chloroform) to extract several phenolic and polyphenolic compounds from three species of the *Combretum* genus in a search for anti-mycobacterial activity. Active extracts were analyzed by ultrahigh-pressure liquid chromatography coupled to quadrupole time-of-flight mass spectrometry (UHPLC/QTOF–MS) and GC–MS. The presence of ellagitannins, gallotannins, ellagic acid derivatives, stilbenoids (particularly dihydrostilbenoids), and many other phenolic compounds was revealed, providing more inputs for pharmaceutical library collections.

Along with the in vitro studies of natural compounds that are reported in the three research papers above, one study examined in vivo effects of the extract from the plant *Epimedium brevicornum* in rats [7]. Other plant extracts were also investigated, including a study on the phytochemical profile and antioxidant activities of *Sphaerostephanos unitus* extracts [8]. The phytoconstituents and pharmacological activities of another plant, wormwood, and its extracts were also reviewed [9]. 

In addition to extracting secondary metabolites from plants and microorganisms, another strategy to produce new compounds with antibacterial properties is via synthesis. Ferrera-Suanzes et al. [10] reported a synthetic approach to obtain degraded analogs of limonoids, which is a group of plant secondary metabolites with several members that show antibacterial activity against multidrug-resistant bacterial strains. This scheme would allow synthesizing molecules with simple structures while preserving the desired properties of limonoids. The research group produced six compounds, and then tested the antimicrobial activities of their stereoisomer mixtures against *Staphylococcus aureus* and its methicillin-resistant strain [10]. 

Another group of natural products with desired bioactive properties is that of nonribosomal peptides. This group notably includes desotamides, which are newly discovered antibacterial compounds. Fazal et al. [11] provided a concise review of this peptide family of antibiotics, particularly focusing on their biosynthesis. Antibacterial peptides can also be found in marine organisms such as ascidians [12]. Many ascidian-derived molecules, including polysulfides, meroterpenes, amino alcohols, alkaloids and furanones, besides peptides, display antibacterial activities. The presence and biological activities of more than 100 ascidian-derived secondary metabolites were systematically reviewed by Casertano et al. [12], providing lead candidates for drug discovery programs. In addition to ascidians, marine bacteria are also a rich source of novel natural products. Santos et al. [13] gave an overview of marine bacterial products, particularly those with antimicrobial, antiviral and anticancer properties [13]. The authors also discussed screening methodologies for the detection of bioactive marine bacteria, and they suggested strategies for avoiding the re-isolation and re-characterization of known bioactive compounds. 

In this Special Issue, an intensive list of natural products and their derivatives with antibacterial, antioxidant and anticancer activities is reported in both research and review papers. It is noted that the knowledge on the biological effects of these compounds is mainly limited to in vitro studies. Future investigations on their in vivo effects and pharmacological properties would provide crucial data for the application of these natural products and their derivatives for fighting against infectious diseases, antibiotic resistance and cancer.
